# Are official confirmed cases and fatalities counts good enough to study the COVID-19 pandemic dynamics? A critical assessment through the case of Italy

**DOI:** 10.1007/s11071-020-05761-w

**Published:** 2020-06-26

**Authors:** Krzysztof Bartoszek, Emanuele Guidotti, Stefano Maria Iacus, Marcin Okrój

**Affiliations:** 1 Department of Computer and Information Science, Linköping University, 581 83 Linköping, Sweden; 2 Institut d’Analyse Financière, University of Neuchâtel, Neuchâtel, Switzerland; 3 European Commission, Joint Research Centre, Via E. Fermi 2749, 21027 Ispra, VA Italy; 4 Department of Cell Biology and Immunology, Intercollegiate Faculty of Biotechnology, University of Gdańsk and Medical University of Gdańsk, Gdańsk, Poland

**Keywords:** COVID-19, Coronavirus, R language, Data, 62-07, 68N15

## Abstract

As the COVID-19 outbreak is developing the two most frequently reported statistics seem to be the raw confirmed case and case fatalities counts. Focusing on Italy, one of the hardest hit countries, we look at how these two values could be put in perspective to reflect the dynamics of the virus spread. In particular, we find that merely considering the confirmed case counts would be very misleading. The number of daily tests grows, while the daily fraction of confirmed cases to total tests has a change point. It (depending on region) generally increases with strong fluctuations till (around, depending on region) 15–22 March and then decreases linearly after. Combined with the increasing trend of daily performed tests, the raw confirmed case counts are not representative of the situation and are confounded with the sampling effort. This we observe when regressing on time the logged fraction of positive tests and for comparison the logged raw confirmed count. Hence, calibrating model parameters for this virus’s dynamics should not be done based only on confirmed case counts (without rescaling by the number of tests), but take also fatalities and hospitalization count under consideration as variables not prone to be distorted by testing efforts. Furthermore, reporting statistics on the national level does not say much about the dynamics of the disease, which are taking place at the regional level. These findings are based on the official data of total death counts up to 15 April 2020 released by ISTAT and up to 10 May 2020 for the number of cases. In this work, we do not fit models but we rather investigate whether this task is possible at all. This work also informs about a new tool to collect and harmonize official statistics coming from different sources in the form of a package for the R statistical environment and presents the “COVID-19 Data Hub.”

## Introduction

In December 2019, the first cases of pneumonia of unknown etiology were reported in Wuhan city, People’s Republic of China. Analyses of patients’ samples collected from their respiratory tract revealed that a novel coronavirus, later named as “severe acute respiratory syndrome coronavirus 2” (SARS-CoV-2) is the pathogen responsible for infection [12]. The disease, officially called COVID-19 by World Health Organization (WHO), is characterized by higher transmissibility and infectivity but lower mortality than Middle East Respiratory Syndrome (MERS) and Severe Acute Respiratory Syndrome (SARS) caused by other coronaviruses [25].

Apart from the source of infection, the spread of the virus depends on the transmission route and general susceptibility of the population. SARS–CoV–2 is believed to be transmitted mostly by close contact (and further carry–over to the mucous surfaces of the body) and inhalation of aerosol produced by an infected person. The presence of the virus was also reported in samples from the gastrointestinal tract [28], but the potential role of the oral–fecal route of infection is unknown. The evidence of asymptomatic carriers who may unintentionally transmit the virus together with relatively long incubation period up to 24 days [2] increase the risk of viral spread worldwide and make prevention measures difficult. On the other hand, separation of identified cases, prior immunity to SARS–CoV–2 or cross-reactivity of human antibodies naturally risen against other viruses would act as a barrier for virus transmission. The latter is probable as RNA sequences of SARS–CoV–2 are in $79\%$ identical to the sequences of SARS–CoV responsible for the previous pandemic in Far East countries in 2002 and $50\%$ identical to MERS–CoV [14]. All above-mentioned issues would act as confounding factors for any modeling of pandemic progression.

Except of the city of Wuhan where the first reports of COVID-19 were announced in December 2019, there was another outbreak of disease, which took place in January–February 2020 on the *Diamond Princess* cruise ship with more than 3700 people onboard. As such a great number of people were locked in a confined space using common facilities, air-condition systems, restaurants, etc., and once the chronology of infections, symptoms and undertaken health measures are known [16, 20, 29], one can consider this as a unique, naturally occurring epidemiological study useful for prediction of mortality, disease spread and other parameters of the COVID-19 pandemic. Since the virus has spread across the world and new pandemic epicenters like Italy, Spain, Iran, South Korea and USA have emerged, a multitude of new data has appeared. Different countries have applied different strategies of testing people for the coronavirus (mass testing vs. testing of selected patients), different testing methods (serological vs. PCR–based assays) and count of case fatalities (solely SARS–CoV–2 positive tested cases vs. cases with comorbidities). Therefore, any direct comparison of pandemic dynamics is difficult but still, comparison to a “golden standard,” which the Diamond Princess case could be considered as, may be useful.

**Fig. 1:**
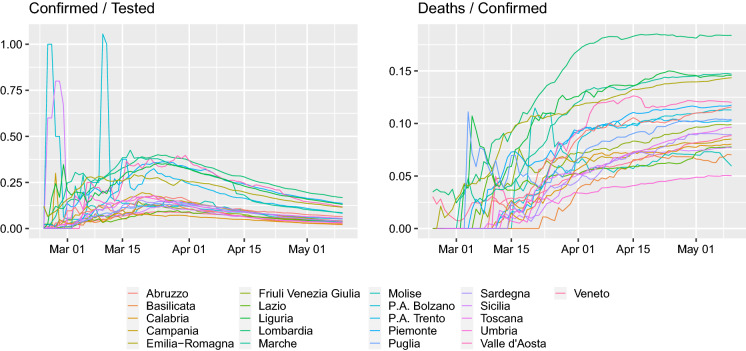
Cumulative confirmed cases and case fatalities for all the regions of Italy. Right: Cumulative case fatalities divided by confirmed cases, left: cumulative confirmed cases divided by the cumulative number of tests

Since the outbreak of the disease, a multitude of papers modeling the dynamics of the infection have appeared, especially on the arXiv preprint server. They are usually concerned with connecting the pandemic with various epidemiological models (e.g., [13, 15, 21, 31] following a brief survey of arXiv at the start of April 2020). However, such models of course require data concerning the infected individuals. Furthermore, the media are bombarding today with two basic numbers (for each country)—the number of confirmed cases and the number of case fatalities. Given that supposedly the vast majority of people are asymptomatic and testing is not done as random sampling of the population but due to particular protocols these values by themselves might be misleading. We can only second [26] in *Despite millions of tests having been performed, there are still no results from statistically well founded sampling-based testing programmes to establish basic epidemic quantities such as infection fatality rate and infection rates. In the absence of such direct data, epidemic management has to proceed on the basis of data produced largely as a side effect of the clinical response to the disease.* As a motivating example, we present Fig. 1 from which we can see that in Italy the case fatality to confirmed ratio is constant, while the confirmed cases to number of tests has been decreasing since around March 22. Indeed, the time period since March 22 is longer than the median time of 19.5 days of infection till death [30], so one should already start observing some drop in the case fatality to confirmed ratio.

Through the case of Italy, this paper tries to investigate the following issues:

- With each country having their own reporting standard and testing strategy are these raw numbers comparable across countries?
- Do these data actually mean what they are being said to be and are they appropriate for model fitting at all?

Clearly, the curves presented in Fig. 1 suggest that a more in-depth look at the raw numbers is required and that there is a need to put the data in a correct perspective before trying to fit any epidemiological model to them, especially because the viral dynamics are starting to be inferred from reported case fatalities [5, 18, 24].

In this work, we approach these issues by looking in detail at the available infection data for individual Italian regions (Sect. 2) and present the R [19] package **COVID19** (Sect. 3) that unifies COVID-19 datasets across different sources in order to simplify the data acquisition process and the subsequent analysis. Section 4 contains a discussion on what other data would be useful (if of course possible to collect for the already overworked public services), in understanding the dynamics of the pandemic. Most regional analyses are contained in the Appendices.

## Italian regional data analysis

Italy is a country which is being very extremely hard–hit with the COVID-19 pandemic. It is currently (as of 13 May 2020) as a whole in lockdown and the medical services are extremely strained. However, due to this situation it has also very detailed epidemiological data that has been made publicly available. Its constantly increasing infected and case fatality count has lead us looking in greater detail into this data, especially as it is used for curve–fitting of epidemiological models (e.g., [13, 15, 21, 31] following brief survey of arXiv) and presented in public media.

The first hurdle that one comes across is what do the presented counts actually represent. This seems to be region dependent.^1^ Furthermore, any deceased whose test result is found positive is classified as a COVID-19 case fatality, regardless of any past or underlying diseases, and this methodology has been consistently applied in Italy since the beginning [17]. It is important to point out that different countries seem to have different testing strategies and classification systems of deaths—hence, raw counts between countries might not be comparable. Given the huge amount of tests performed in Italy 2,735,628 (as of 13th May 2020, [11]) an important question is: “*what fraction of them were serological tests?*” as there is no official data on this. A serological test may not distinguish between a person actively infected with the virus and a person that was exposed to the virus in the past. Alternatively, serological test may not detect person actively infected with still low viral titer of anti-virus antibodies. On the other hand, if the protocol is to test only people exhibiting symptoms and medical personnel, then given that it is hypothesized that the vast majority of cases are asymptomatic, such a raw count might not be representative of the scale of the epidemic.

Given the above uncertainties, we set out to see how the Italian regional data could be presented in a standardized manner. Furthermore, we see how the data of each region compares to the *Diamond Princess’* data. We focus on the two values that are being presented everywhere—the confirmed case count and the case fatalities count. However, these should be scaled. We scale the confirmed case count by the total number of tests performed. Scaling the case fatalities is more problematic. A common way is to present them as the case fatality ratio, but these may be misleading when estimated during an epidemic [4]. Furthermore, assuming that the vast majority of cases are asymptomatic—hence, not tested and not inside the case count, we are uncertain to what the fatalities would actually be compared to.

Given the lack of hard data, another objective approach would be to compare the daily count of case fatalities to the total deceased count for the day. To the best of our knowledge, such statistics are not centrally reported in Italy in real time. Daily deceased counts (from nearly all of the Italian municipalities—see Discussion) are available though for the period 1 January–15 April 2020.^2^ Hence, for this time period we are able to plot the weakly “nearly”-desired ratios (see Sect. 4). We aggregate per week to remove daily fluctuations, which obscure the picture. Furthermore, the same data source provides deceased counts for the years 2015–2019 (for the same time period). This allows us to also visualize the excess mortality (with respect to the per week average from the past five years). Beyond this time interval, it is impossible to provide such curves. However, having daily case fatalities counts and past mortality (this is taken as a constant value equaling the average number of deceased for 15th April) we are able to plot the (per week) ratio of case fatalities to previous average mortality. This provides some indication of the magnitude of excess mortality.^3^ However, it is worth noticing that when looking at the current excess mortality it could be appropriate to compare with past mortality peak (e.g., for UK death toll, the 2014/2015 and 1999/2000 peaks,^4^ Figures 1, 5 and 6 of [23]), taking into consideration the causes of death. Here for Italy and its regions, in Figs. 4, 6 and “Figs. 28, 29, 30, 31, 32 in Appendix B” we compare the current deceased peak with the seasonal start of the January one.

We should remark that perhaps more focus should be on the cumulative positive test fraction instead of the daily positive test fraction. This is because the daily fraction is extremely noisy and furthermore, it sometimes happens that this fraction, in the official data source for Italy, exceeds 1. For similar reasons, we plot the weekly scaled deaths and cumulative scaled deaths. The daily counts are extremely noisy as well.

We plot the scaled daily and cumulative positive test count and scaled case fatalities next to the cumulative positive tested fraction of passengers on the *Diamond Princess*. Here, we present the graphs from two special regions in Italy: Lombardy and Veneto. The remaining regions are presented in “Appendix A.” Lombardia is the center of the epidemic, where the cases and deaths counts are the highest. Veneto seems to be a region where the pandemic’s dynamics are special. It was a region that very early on undertook population-wide testing and drastic lockdown measures.^5^

**Fig. 2:**
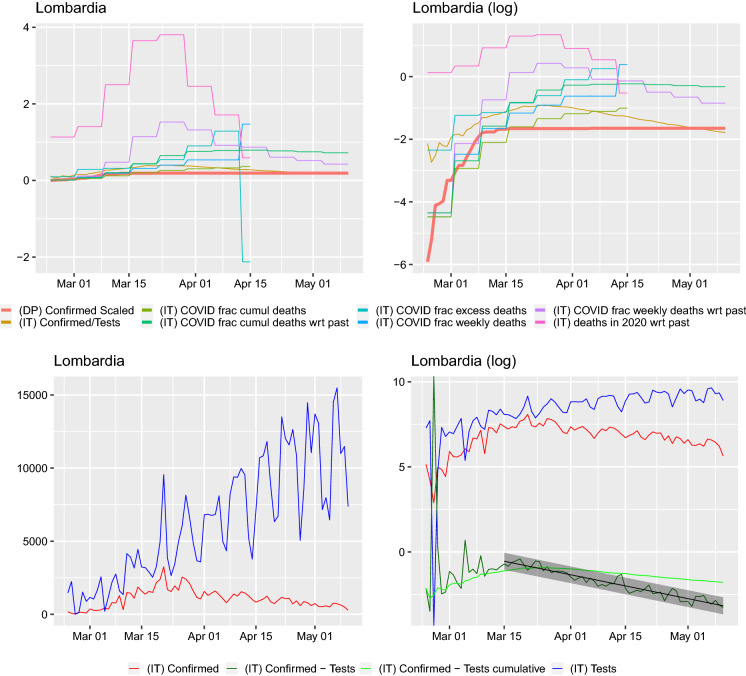
Comparison of curves for Lombardy region. Left: *y*–axis on normal scale, right: on logarithmic scale. Regression line shown for $\log (\mathrm {daily~confirmed})-\log (\mathrm {daily~tested})\sim time$ with $95\%$ prediction band. Slope of regression with $95\%$ confidence interval: $a_{\mathrm {f}}=-0.047 (-0.051,-0.043)$, this corresponds to a half-life (in days) of 14.829(13.712, 16.144). The slope of the regression $\log (\mathrm {daily~confirmed})\sim time$ is $a_{\mathrm {raw}}=-0.025 (-0.029,-0.021)$ corresponding to a half-life (in days) of 27.656(23.961, 32.698). The slope of the regression $\log (\mathrm {daily~tests})\sim time$ is 0.022(0.016, 0.030) corresponding to a doubling time (in days) of 31.970(23.280, 42.506). Ratio of slopes for $a_{\mathrm {f}}/a_{\mathrm {raw}}=1.865$, with corresponding half-lives’ ratio: 0.536. The slope of the regression $\log (\mathrm {cumulative~confirmed})-\log (\mathrm {cumulative~tested})\sim time$ is $-0.016 (-0.018,-0.015)$ corresponding to a half-life (in days) of 42.525(39.386, 46.208)

**Fig. 3:**
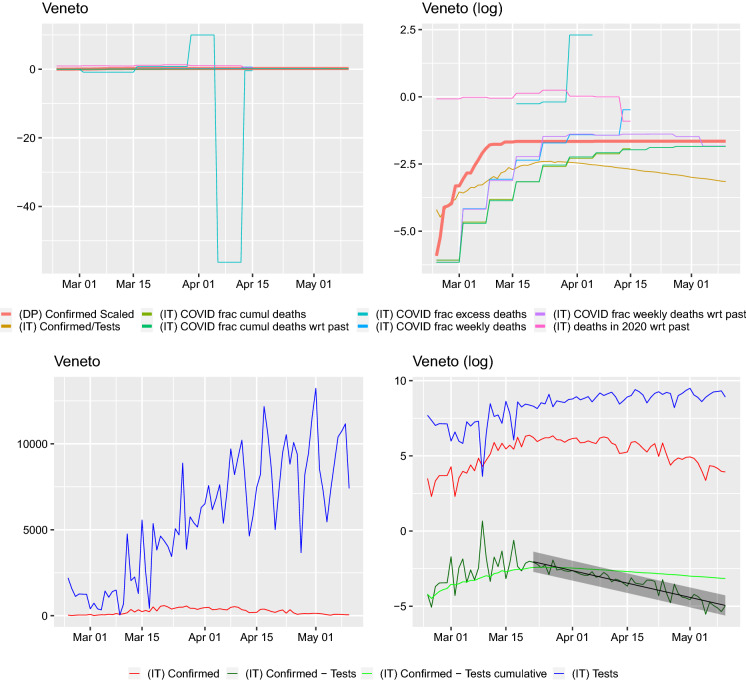
Comparison of curves for Veneto region. Left: *y*–axis on normal scale, right: on logarithmic scale. Regression line shown for $\log (\mathrm {daily~confirmed})-\log (\mathrm {daily~tested})\sim time$ with $95\%$ prediction band. Slope of regression with $95\%$ confidence interval: $a_{\mathrm {f}}=-0.059 (-0.065,-0.053)$ corresponding to a half-life (in days) of 11.693(10.592, 13.049). The slope of the regression $\log (\mathrm {daily~confirmed})\sim time$ is $a_{\mathrm {raw}}=-0.047 (-0.054,-0.040)$ corresponding to a half-life (in days) of 14.843(12.879, 17.513). The slope of the regression $\log (\mathrm {daily~tests})\sim time$ is 0.013(0.007, 0.026) corresponding to a doubling time (in days) of 55.100(26.467, 95.637). Ratio of slopes for $a_{\mathrm {f}}/a_{\mathrm {raw}}=1.269$, with corresponding half-lives’ ratio: 0.788. The slope of the regression $\log (\mathrm {cumulative~confirmed})-\log (\mathrm {cumulative~tested})\sim time$ is $-0.016 (-0.017,-0.016)$ corresponding to a half-life (in days) of 42.325(40.947, 43.799)

**Fig. 4:**
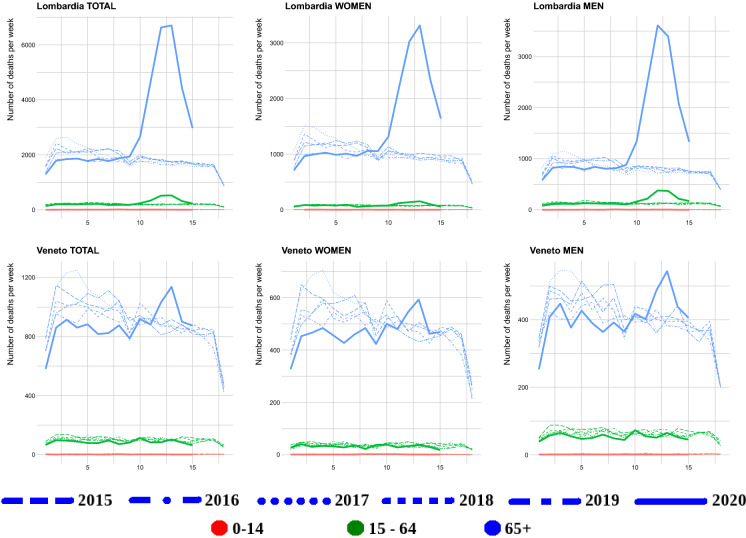
Weekly raw death toll comparison in different age groups between 2020 and 2015–2019 for Lombardy and Veneto

On all of the graphs, the curve labels have the following meaning.

(DP) Confirmed Scaled: cumulative number of cases on the Diamond Princess divided by 3711, the number of passengers and crew onboard
(IT) Confirmed/Tests: cumulative confirmed case to cumulative number of tests ratio for Italy or region
(IT) COVID frac cumul deaths: cumulative number of case fatalities to cumulative number of deceased in 2020 ratio for Italy or region
(IT) COVID frac cumul deaths wrt past: cumulative number of case fatalities to cumulative number of average from 2015–2019 number of deceased ratio for Italy or region
(IT) COVID frac excess deaths: number of case fatalities for given week to difference between deaths in 2020 and average from 2015–2019 number of deceased for given week ratio for Italy or region
(IT) COVID frac weekly deaths: number of case fatalities for given week to number of deceased in 2020 for given week ratio for Italy or region
(IT) COVID frac weekly deaths wrt past: number of case fatalities for given week to average from 2015–2019 number of deceased for given week ratio for Italy or region
(IT) deaths in 2020 wrt past: number of deceased in 2020 for given week to average from 2015–2019 number of deceased for given week ratio for Italy or region
(IT) Confirmed: daily number of confirmed cases for Italy or region
(IT) Confirmed - Tests: $\log ($ (IT) Confirmed ) - $\log ($ (IT) Tests )
(IT) Confirmed - Tests cumulative: $\log ($ cumulative number of confirmed cases in Italy or region ) - $\log ($ cumulative number of tests performed in Italy or region )
(IT) Tests: daily number of tests performed for Italy or region

We obtain data for the period 24 February–10 May 2020, and we plot the curves from the moment of the first death. From both Figs. 2 and 3 (and those present in the “Appendix A”), we can notice a number of facts. Firstly, the daily fraction of infected cases fluctuates very wildly and sometimes can be greater than 1. This can only be due to some changes in protocols or reporting. Similarly, such an explanation seems plausible for the fluctuations in the fractions. In fact, [17] report a change in the way positive cases and deaths are calculated on 10 March. The cumulative case fraction on the other hand does not exhibit such fluctuations. For most regions, it is flat and then decreasing. In a number of regions (e.g., Abruzzo, Basilicata, Campania, Friuli Venezia Giulia, Lazio, Molise, Puglia, Sardegna, Sicily, Toscana, Umbria, Veneto) on the log–scale graphs, the cumulative case to tests ratio curve seems the peak around or below the *Diamond Princess*’ cumulative case curve and then start dropping. The scaled death curves exceed this curve.

When looking at the graphs of the number of tests per day, two things can be seen. Firstly, the number of positive cases closely follows the number of tests (this is clearly visible on the log–scale graphs and supported by the regression study). We look at this issue in detail and present for each region and Italy the confirmed cases with respect to the total tests carried out. We also regress the $\log ($(daily confirmed cases)/(daily total tests)) on *time* (in days) and $\log ($(cumulative confirmed cases)/(cumulative total tests)) on *time* (in days). The slope of such a regression can be presented in terms of the half-lives, if it is negative. Such a presentation in terms of effect sizes is important; otherwise, it is difficult to assess if the raw slope is big or small. The linear model approach means that the proportion of infected behaves exponentially

$$\begin{aligned} \frac{\mathrm {Daily(cumulative)~confirmed~cases}}{\mathrm {Daily(cumulative)~total~tests}} =: p(t) = b e^{at}, \end{aligned}$$

then to get the half-life (for *a* negative, $t_{2}>t_{1}$) one takes

$$\begin{aligned} 2=\frac{p(t_{2})}{p(t_{1})}=e^{a(t_{2}-t_{1})} \end{aligned}$$

obtaining $(t_{2}-t_{1})=\log (2)/(-a)$. For $a>0$, one will obtain the doubling time in the same way as $(t_{2}-t_{1})=\log (2)/a$. It is important to point out that this is a rather rule–of–thumb approach—our aim is *not* to model the dynamics of infections, but rather to visualize and understand what the data in front of us are. These regressions were not performed from the first day, as initially there seems to be a lot of noise in the tests, the starting time considered is visible in each graph—where the fitted line with prediction confidence band is fitted. We performed a regression for both the daily and cumulative counts. For some regions (Molise, Valle d’Aosta), no regression is performed as the daily counts seem to noisy. Secondly, one can very clearly identify days when something must have changed due to the testing methodology in the Emilia Romagna region—there are huge dips in the numbers of tests performed. Hence, for this region the dates 28–30 March were removed for the regression estimation. In the Basilicata and Calabria regions, spikes to 0 can also be observed (these are also removed, as on the log scale would result in infinite values which cannot be handled by the regression procedure in lm(). However, such dips require careful investigation.

**Fig. 5:**
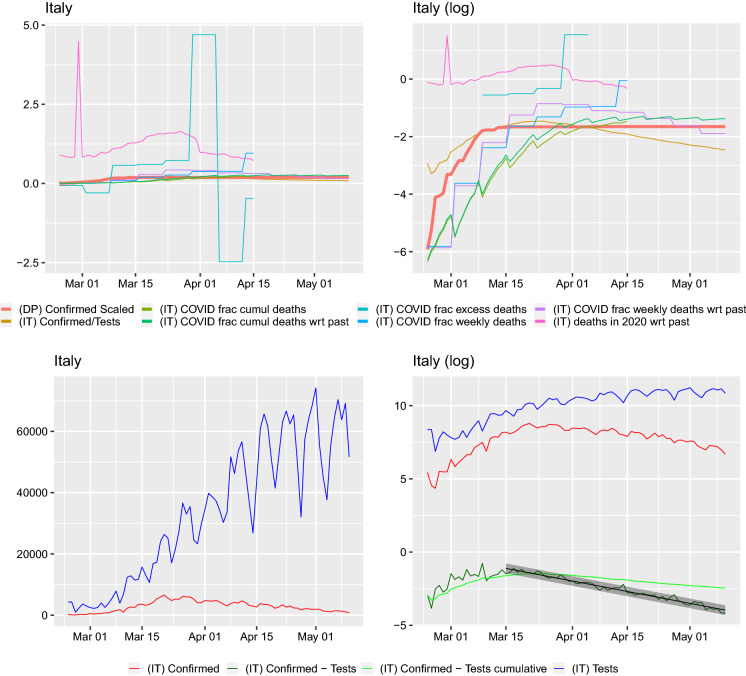
Comparison of curves for the whole of Italy. Left: *y*–axis on normal scale, right: on logarithmic scale. Regression line shown for $\log (\mathrm {daily~confirmed})-\log (\mathrm {daily~tested})\sim time$ with $95\%$ prediction band. Slope of regression with $95\%$ confidence interval: $a_{\mathrm {f}}=-0.051 (-0.053,-0.048)$ corresponding to a half-life (in days) of 13.617(12.972, 14.330). The slope of the regression $\log (\mathrm {daily~confirmed})\sim time$ is $a_{\mathrm {raw}}=-0.027 (-0.031,-0.023)$ corresponding to a half-life (in days) of 25.631(22.348, 30.046). The slope of the regression $\log (\mathrm {daily~tests})\sim time$ is 0.024(0.020, 0.036) corresponding to a doubling time (in days) of 29.052(19.224, 35.217). Ratio of slopes for $a_{\mathrm {f}}/a_{\mathrm {raw}}=1.882$, with corresponding half-lives’ ratio: 0.531. The slope of the regression $\log (\mathrm {cumulative~confirmed})-\log (\mathrm {cumulative~tested})\sim time$ is $-0.019 (-0.020,-0.018)$,this corresponds to a half-life (in days) of 35.882(33.884, 38.130)

The directly plotted death toll in Figs. 4, 5, 28, 29, 30, 31 and 32 shows that in the regions Emilia-Romagna, Lombardia and Valle d’Aosta, P.A. Bolzano combined with P.A. Trento, there is a larger current (spring 2020) mortality peak than the past December/January (2015–2020 are plotted separately) maximum one. In the regions Liguria, Marche and Piemonte, such a larger current peak is present for men only. In the other regions for all age groups and both men and women, the current “COVID-19 peak” seems to be approximately of the same height, or lower, than past December/January (2015–2019) maximum ones. Looking at Italy for men and both sexes combined, it is higher, but women seem to have the same peak height. However, it must be stressed that this is only considering the peak’s height, not the total amount of deceased during the current peak and the December/January ones.

## **COVID-19**R package

We used the, available on CRAN, **COVID19** R package for the purpose of obtaining the data.^6^ The package unifies COVID-19 datasets across different sources in order to simplify the data acquisition process and the subsequent analysis. COVID-19 data are pulled in real time and merged with demographic indicators from several trusted sources including but not limited to: Johns Hopkins University Center for Systems Science and Engineering (JHU CSSE);^7^ World Bank Open Data;^8^ World Factbook by CIA;^9^ Ministero della Salute, Dipartimento della Protezione Civile;^10^ ISTAT - Istituto Nazionale di Statistica;^11^ Swiss Federal Statistical Office;^12^ Open Government Data Zurich.^13^ Besides worldwide data, the dataset includes fine-grained data for the *Diamond Princess*, Switzerland and Italy. At the time of writing, these include the number of confirmed cases, deaths and tests, total population, population ages 0–14, 15–64 and $65+$ (% of total population), median age of population, population density per km$^{2}$, population mortality rate. Depending on the data provider, the data are available at the country level, state level, or city level. For non-R users, the combined datasets are available in csv format.^14^

## Discussion or Should we use these data to calibrate epidemiological models?

In this work, we analyzed in depth the two statistics that are commonly reported for the currently ongoing COVID-19 pandemic—the number of confirmed cases and the number of case fatalities for the different regions of Italy. We found significant variability between regions but also some common insights. In particular, the number of confirmed cases is clearly related to the number of tests and their ratio seems to be decaying for some time now in all regions. This is confirmed when looking at the log–scale plot. The difference between the logarithm of the cumulative number of tests and the logarithm of the cumulative number of confirmed seems to be (visually) dropping linearly (apart from the below, extremely noisy ones) regions and Italy as a whole. Furthermore, for a number of regions (Molise, Valle d’Aosta), on the log scale, the tests, total, positive and difference behave very chaotically, suggesting rather various test handling situations, than any pattern. Such oscillations can be visible in all regions at the initial stages, but they settle down (apart from the previously mentioned three regions). However, in regions with seemingly well-behaved curves individual huge dips can be observed (Emilia-Romagna, Marche). Therefore, reports claiming the growth of the epidemic based only on the increasing number of confirmed individuals will not be catching its dynamics.

Furthermore, studying only daily positively tested counts could be misleading. On a number of days, we found (for some regions) that this count was greater than the number of tests performed. This can certainly be understood, as the result of reporting procedures, in a crisis situation. However, this also implies that any statistical analysis or modeling of such data has to be done very carefully. We find that the cumulative positively tested fraction behaves much more stably, even though in the official cumulative counts decreases can be observed.

More importantly, using the raw confirmed case counts one could risk combining the sampling effort with the actual disease spread. In our regressions, for the logarithm of the ratio confirmed cases to total tests on time the fitted slopes are all negative (indicating that the virus is receding and this was observed also by [7]).

Furthermore, these slopes are steeper than the slopes of the logarithm of the raw confirmed case counts on time. With the exceptions of Lazio, P. A. Bolzano the $95\%$ confidence intervals for these two slopes do not overlap, or overlap very slightly. The ratios of the two slopes lie between 1.176 (P. A. Bolzano) and 3.717 (Piemonte). We report these ratios alongside the slope estimates in the captions of Figs. 7, 8, 9, 10, 11, 12, 13, 14, 15, 16, 17, 18, 19, 20, 21, 22, 23, 24, 25, 26 and 27. This means that the number of confirmed cases will be confounded by the number of performed tests and cannot be analyzed without them as a point of reference.

Hence, the raw confirmed case counts are not representative of the virus’ infection dynamics. The logarithm of the fraction of confirmed cases to total tests is modeled well by a linear function with an increasing number of daily tests being performed and has a steeper slope than the logarithm of the confirmed case counts. Drawing conclusions from raw confirmed case data would seem to be mixing–in the study of the sampling effort (it is important to stress that we do not make any statements here concerning the interpretation of the confirmed cases to tests fraction). Therefore, calibrating model parameters for this virus’s dynamics should not be done based solely on confirmed case counts, but maybe rather also on case fatalities or hospitalization data (given that classification protocols are taken into account) as, e.g., [5, 18, 24] do. In fact, already [9], critised (as [18] later also did following them) looking at case counts and postulated a focus on the “observed deaths” while [24] writes that “the cumulative number of deaths can be regarded as a master variable.” [6] developed an estimation methods based on the cumulative reported number of case fatalities.

On the other hand, we also looked at the ratio of case fatalities to the number of deceased per day. This has the analytical advantage, of referring to something certain and well measured, and detailed records are collected (sooner or later) on the exact number of deceased in a given time period. Here, there is hardly any chance of missing asymptomatic (of being dead) people. *If the assumption*, mentioned in the Introduction, that a significant proportion of the tests is serological is true, then the ratio of case fatalities to all deceased should be telling us something about the cumulative proportion of infected individuals. Our graphs (especially on the log–scale) do not contradict this, while the cumulative proportion of confirmed cases changes very slowly, the ratio of case fatalities to total deceased per day seems to look like an epidemic growth curve. Since Italy has very high-quality data on the case fatalities, this data could be further studied to assess the dynamics of the pandemic (e.g., [15] uses the raw death counts for assessing the dynamics of the pandemic, albeit at the country level). This seems to be supported by that if one compares the curves to a potential “gold standard”—the cumulative fraction of confirmed cases on the *Diamond Princess*, then the case fatalities ratio seems to shadow this curve (on the initial part when the epidemic was taking place on the cruise ship and for some regions like Emilia–Romagna or Lombardia) but exceeds it. One could hope that once all curves would flatten at the same level, then the epidemic will reach the plateau. Unfortunately, at the level of some (e.g., Emilia–Romagna or Lombardia) of the regions, the scaled case fatalities grew and exceeded both the *Diamond Princess* and cumulative fraction of confirmed cases.

**Fig. 6:**
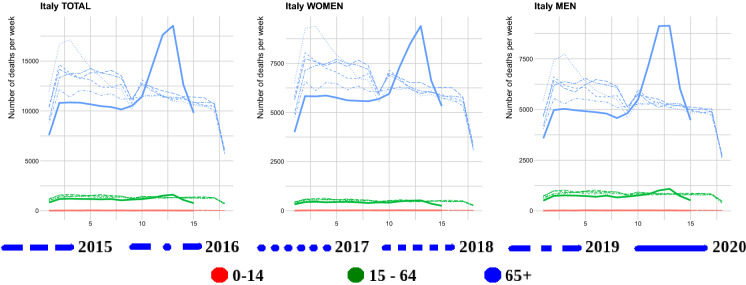
Weekly raw death toll comparison in different age groups between 2020 and 2015–2019 for whole of Italy

**Fig. 28:**
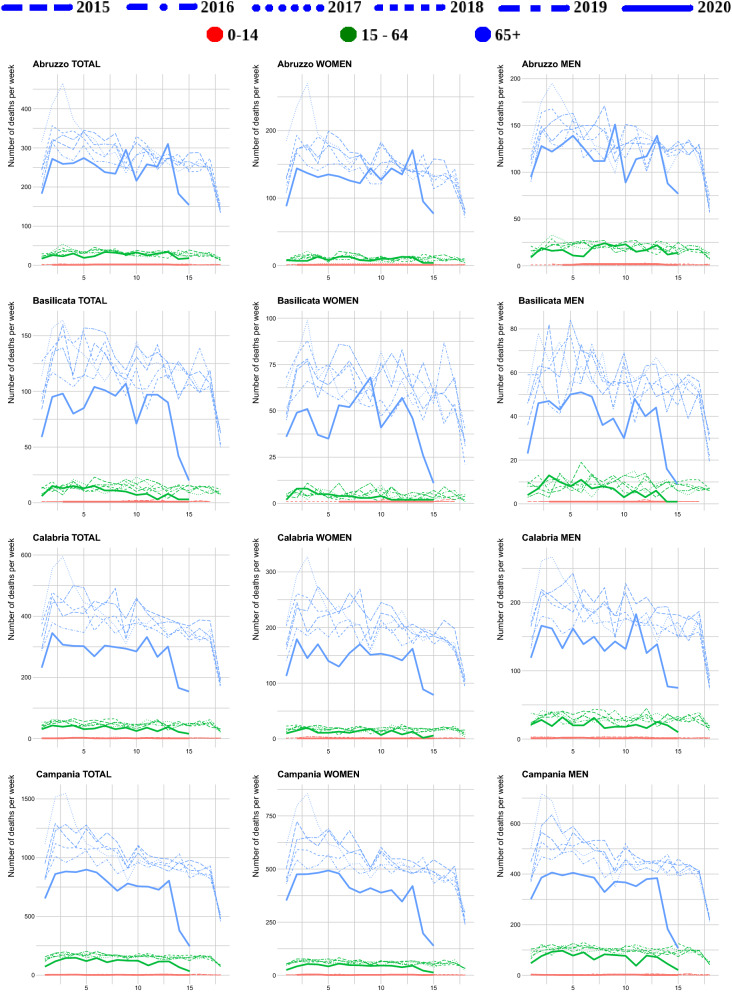
Weekly raw death toll comparison in different age groups between 2020 and 2015–2019 for regions: Abruzzo,Basilicata, Campania and Calabria

**Fig. 29:**
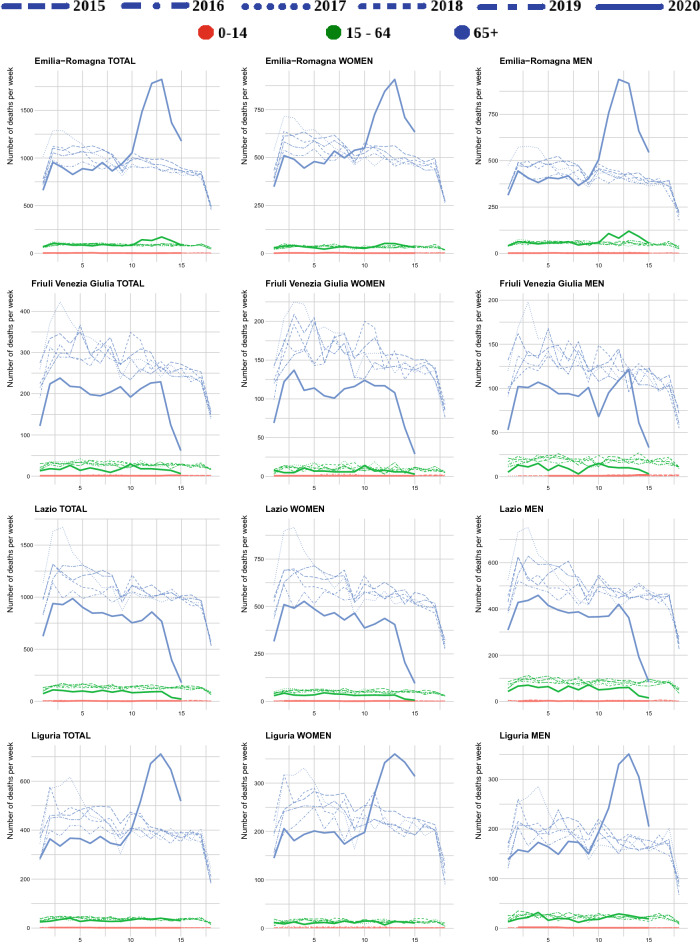
Weekly raw death toll comparison in different age groups between 2020 and 2015–2019 for regions: Emilia Romagna, Friuli Venezia Giulia, Lazio and Liguria

**Fig. 30:**
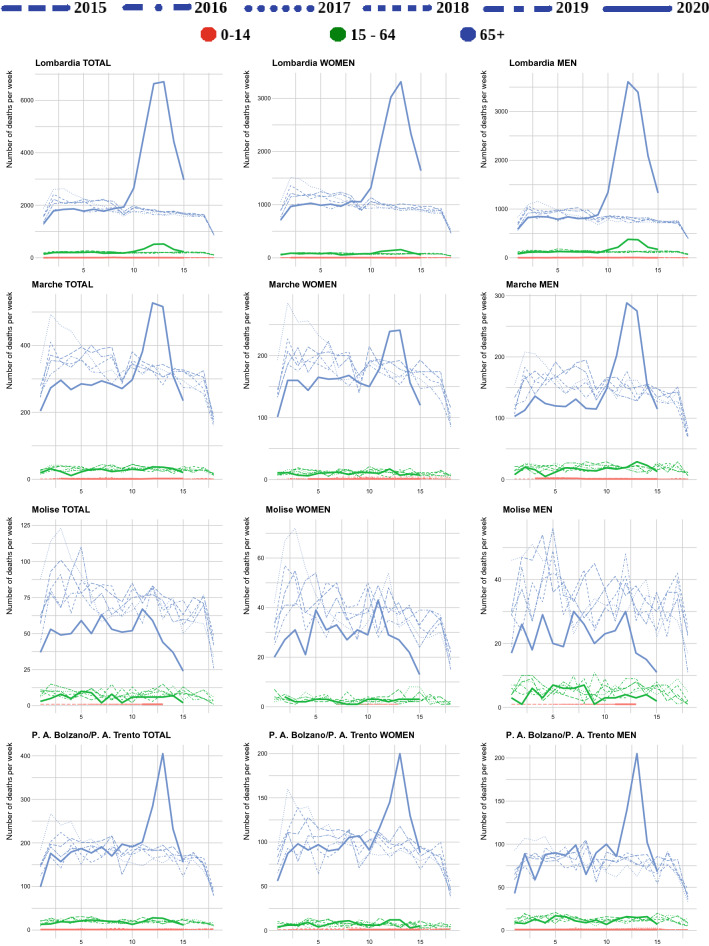
Weekly raw death toll comparison in different age groups between 2020 and 2015–2019 for regions: Lombardia, Marche, Molise and P.A. Bolzano and P.A. Trento

**Fig. 31:**
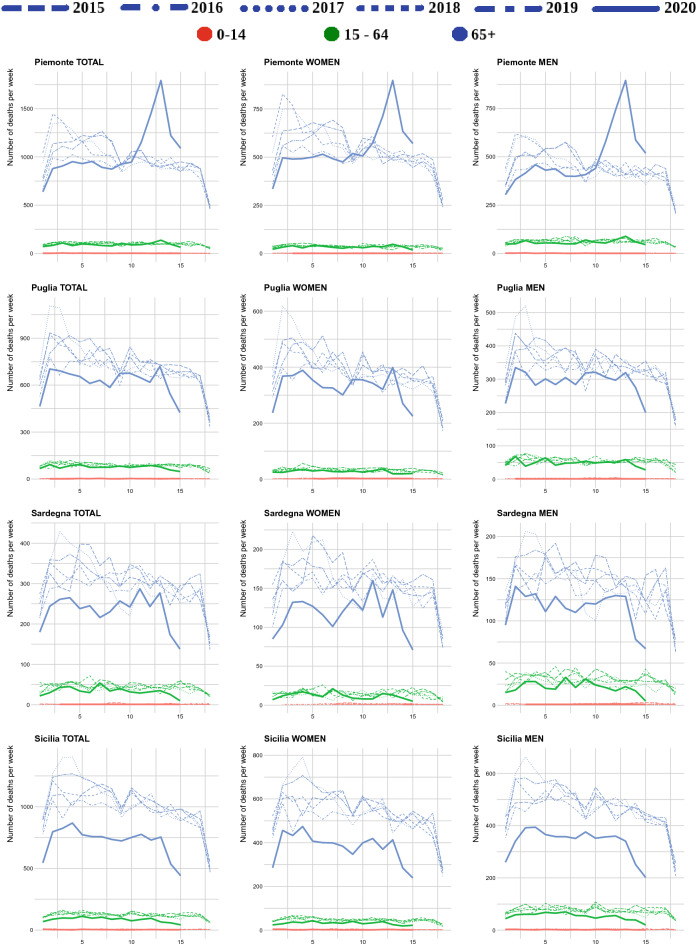
Weekly raw death toll comparison in different age groups between 2020 and 2015–2019 for regions: Piemonte, Puglia, Sardegna and Sicilia

**Fig. 32:**
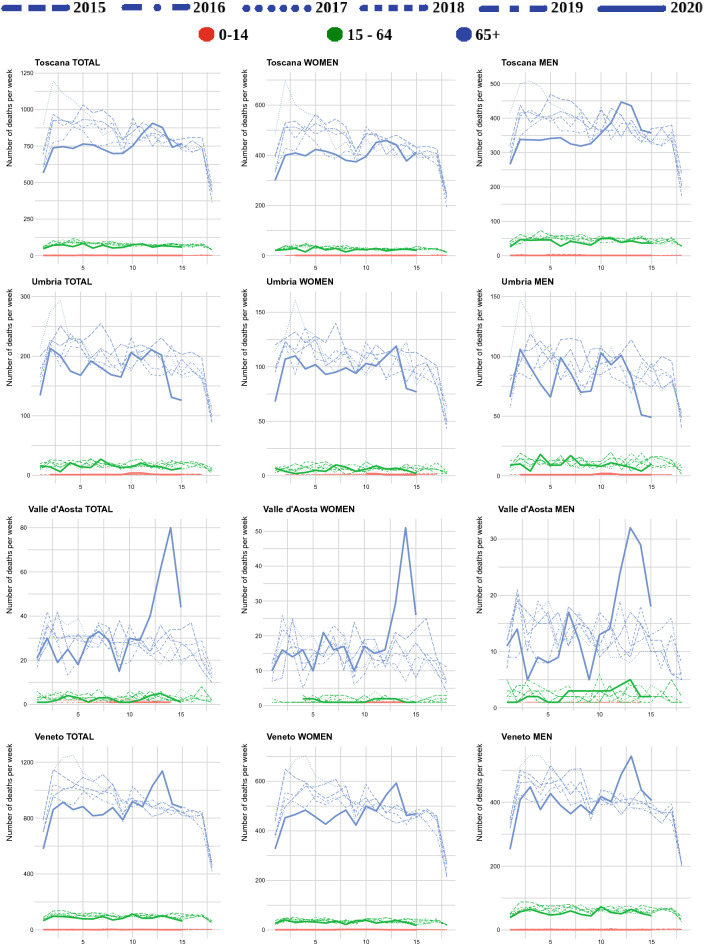
Weekly raw death toll comparison in different age groups between 2020 and 2015–2019 for regions: Toscana, Umbira, Valle d’Aosta and Veneto

We also compared the regional results to the same curves for the whole of Italy, Fig. 5. On the one hand, the same patterns are visible—the number of confirmed cases are related to the testing effort, the case fatalities exceeding the *Diamond Princess*’ cumulative confirmed cases and the confirmed cases fraction seems to be stabilizing around the *Diamond Princess*’ and then dropping. However, these graphs completely miss the regional variation. This is particularly visible when looking at the total death tolls directly Figs. 6, 28, 29, 30, 31 and 32. Combined Italy shows a visible increase in the death toll during the March–April period compared to previous years and the seasonal December/January peak. However, this peak is driven by particular regions Emilia Romagna, Lombardia and Piemonte (Liguria, Marche, Valle d’Aosta, P.A. Bolzano combined with P.A. Trento also shows a big increase, but in raw numbers are much lesser than the other three). All the other regions’ peak is on the same level or lower than the December/January one and for some the death toll is on similar levels to the March–April one from previous years. Furthermore, looking just at epidemiological country level data would be especially misleading for Italy as Lombardy acted differently from Veneto in terms of their testing strategies.

We believe that our presented view on the Italian regional data gives some insights how the pandemic data reporting can be improved (if of course given the difficult situation, it would be possible in practise). For the confirmed cases count, a breakdown should be provided, how many of these were medical personnel, how many had symptoms, how many were seriously hospitalized before, how many were tested for other reasons (e.g., after contact). Similarly for the number of tests carried out and their type (serological or not). The case fatalities counts should also be put in perspective with a report of how many people died in total on the given day and how many deceased were tested negatively. This would allow for estimating excess mortality (crudely compared to previous years’ average or more exactly if number of deaths for the given time period are available) and for correct scaling to compare to other ratios. In fact, in the time period 1 January–15 April, we are able to visualize the excess mortality directly—the number of deceased (in each week) in 2020 to the average from the past five years. The dataset is based on the 7,904 Italian municipalities.

To the best of our knowledge, the presented here counts are at the moment the best available data that can be used for scaling and putting the deceased counts in Italy in perspective. The death counts seem to be collected in a consistent manner, both the number of case fatalities and the (used here) population death counts. This means that such counts could be used as a proxy for monitoring the dynamics of the virus.

It is also a question whether the *Diamond Princess* can be considered as a gold–standard. Certainly at the beginning it seems to behave like the other presented here curves. However, the data very quickly end, when the passengers were disembarked. We do not know if it reached the plateau or would have still grown. The confirmed case ratio seems to usually stay below/around this curve, slightly go above and then drop. Scaled case fatality curves exceed the curve.

Finally, the counting methodology should be made readily available for easy comparison between different countries. While of course each country is free to follow their own protocol, without putting numbers into context one can analyze data in an over-pessimistic or over-optimistic way. The effect of different counting methods is pointed out by [3, 17, 22], when fitting parameters to the confirmed case counts (in Lombardy, Bergamo and Brescia), one has a change of coefficients following 10 March and 17 March, and the latter can be possibly due to containment measures, but the former the authors are convinced is due to a change in the counting methodology. We have also abstained here from fitting any models to the data (the regression performed does not have as an aim modeling but formally testing what the respective curve could be telling us). It is known that due to different protocols between regions and changes in the protocols with time, the data are not homogeneous. In order to fit any model, one would have to obtain documentation what were the measurement strategies for each region in the time periods. In fact, when [1] modeled the cumulative number of infections in Italy through time (obtained using the **COVID19** R package), they performed fits to date separately in different time intervals which corresponded to various government introduced confinement measures.

## Source code, data and scripts

The **COVID19** R data package is available from https://cran.r-project.org/web/packages/COVID19/ . The R script used to generate the graphics is available from https://github.com/krzbar/COVID19 .

## Figures and Tables

**Fig. 7:**
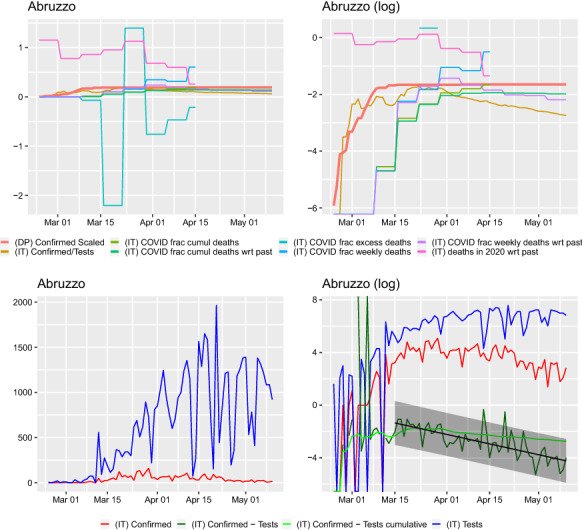
Comparison of curves for Abruzzo region. Left: *y*–axis on normal scale, right: on logarithmic scale. Regression line shown for $\log (\mathrm {daily~confirmed})-\log (\mathrm {daily~tested})\sim time$ with $95\%$ prediction band. Slope of regression with $95\%$ confidence interval: $a_{\mathrm {f}}=-0.051 (-0.064,-0.039)$, this corresponds to a half-life time (in days) of 13.501(10.830, 17.923). The slope of the regression $\log (\mathrm {daily~confirmed})\sim time$ is $a_{\mathrm {raw}}=-0.030 (-0.040,-0.021)$ corresponding to a half-life (in days) of 22.801(17.203, 33.796). The slope of the regression $\log (\mathrm {daily~tests})\sim time$ is 0.021(0.010, 0.048) corresponding to a doubling time (in days) of 33.105(14.398, 68.114). Ratio of slopes for $a_{\mathrm {f}}/a_{\mathrm {raw}}=1.689$, with corresponding half-lives’ ratio: 0.592. The slope of the regression $\log (\mathrm {cumulative~confirmed})-\log (\mathrm {cumulative~tested})\sim time$ is $-0.017 (-0.019,-0.015)$ corresponding to a half-life (in days) of 40.259(35.924, 45.784)

**Fig. 8:**
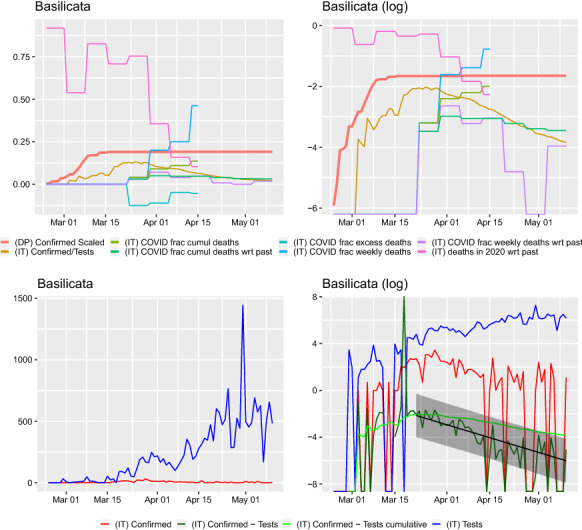
Comparison of curves for Basilicata region. Left: *y*–axis on normal scale, right: on logarithmic scale. Regression line shown for $\log (\mathrm {daily~confirmed})-\log (\mathrm {daily~tested})\sim time$ with $95\%$ prediction band. Slope of regression with $95\%$ confidence interval: $a_{\mathrm {f}}=-0.079 (-0.099,-0.059)$, this corresponds to a half-life (in days) of 8.786(7.037, 11.693). The slope of the regression $\log (\mathrm {daily~confirmed})\sim time$ is $a_{\mathrm {raw}}=-0.036 (-0.054,-0.018)$ corresponding to a half-life (in days) of 19.170(12.816, 38.025). The slope of the regression $\log (\mathrm {daily~tests})\sim time$ is 0.038(0.030, 0.028) corresponding to a doubling time (in days) of 18.446(24.860, 23.491). Ratio of slopes for $a_{\mathrm {f}}/a_{\mathrm {raw}}=2.182$, with corresponding half-lives’ ratio: 0.458. The slope of the regression $\log (\mathrm {cumulative~confirmed})-\log (\mathrm {cumulative~tested})\sim time$ is $-0.041 (-0.043,-0.040)$ corresponding to a half-life (in days) of 16.866(16.265, 17.513)

**Fig. 9:**
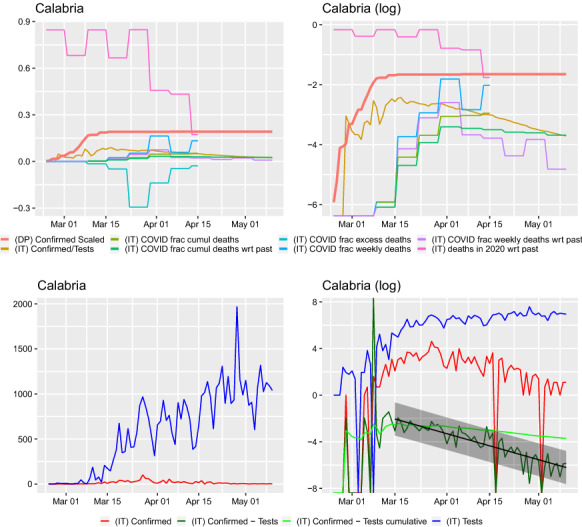
Comparison of curves for Calabria region. Left: *y*–axis on normal scale, right: on logarithmic scale. Regression line shown for $\log (\mathrm {daily~confirmed})-\log (\mathrm {daily~tested})\sim time$ with $95\%$ prediction band. Slope of regression with $95\%$ confidence interval: $a_{\mathrm {f}}=-0.074 (-0.085,-0.063)$,this corresponds to a half-life (in days) of 9.315(8.122, 10.918). The slope of the regression $\log (\mathrm {daily~confirmed})\sim time$ is $a_{\mathrm {raw}}=-0.052 (-0.064,-0.040)$ corresponding to a half-life (in days) of 13.249(10.747, 17.271). The slope of the regression $\log (\mathrm {daily~tests})\sim time$ is 0.022(0.016, 0.043) corresponding to a doubling time (in days) of 31.709(16.233, 42.761). Ratio of slopes for $a_{\mathrm {f}}/a_{\mathrm {raw}}=1.422$, with corresponding half-lives’ ratio: 0.703. The slope of the regression $\log (\mathrm {cumulative~confirmed})-\log (\mathrm {cumulative~tested})\sim time$ is $-0.023 (-0.024,-0.022)$ corresponding to a half-life (in days) of 30.582(29.262, 32.028)

**Fig. 10:**
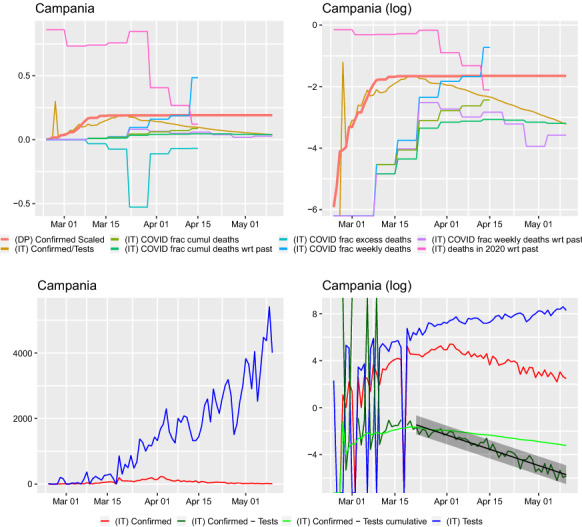
Comparison of curves for Campania region. Left: *y*–axis on normal scale, right: on logarithmic scale. Regression line shown for $\log (\mathrm {daily~confirmed})-\log (\mathrm {daily~tested})\sim time$ with $95\%$ prediction band. Slope of regression with $95\%$ confidence interval: $a_{\mathrm {f}}=-0.087 (-0.094,-0.079)$ ,this corresponds to a half-life (in days) of 7.998(7.376, 8.734). The slope of the regression $\log (\mathrm {daily~confirmed})\sim time$ is $a_{\mathrm {raw}}=-0.055 (-0.062,-0.047)$ corresponding to a half-life (in days) of 12.707(11.155, 14.762). The slope of the regression $\log (\mathrm {daily~tests})\sim time$ is 0.032(0.027, 0.032) corresponding to a doubling time (in days) of 21.580(21.771, 25.624). Ratio of slopes for $a_{\mathrm {f}}/a_{\mathrm {raw}}=1.589$, with corresponding half-lives’ ratio: 0.629. The slope of the regression $\log (\mathrm {cumulative~confirmed})-\log (\mathrm {cumulative~tested})\sim time$ is $-0.031 (-0.032,-0.030)$ corresponding to a half-life (in days) of 22.438(21.964, 22.933)

**Fig. 11:**
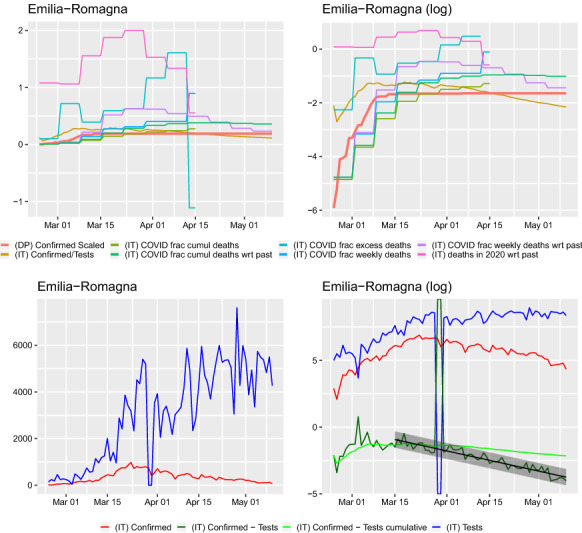
Comparison of curves for Emilia Romagna region. Left: *y*–axis on normal scale, right: on logarithmic scale. Regression line shown for $\log (\mathrm {daily~confirmed})-\log (\mathrm {daily~tested})\sim time$ with $95\%$ prediction band. Slope of regression with $95\%$ confidence interval: $a_{\mathrm {f}}=-0.050 (-0.055,-0.046)$, this corresponds to a half-life (in days) of 13.774(12.594, 15.196). The slope of the regression $\log (\mathrm {daily~confirmed})\sim time$ is $a_{\mathrm {raw}}=-0.034 (-0.038,-0.029)$ corresponding to a half-life (in days) of 20.681(18.199, 23.948). The slope of the regression $\log (\mathrm {daily~tests})\sim time$ is 0.016(0.011, 0.035) corresponding to a doubling time (in days) of 42.042(19.760, 62.087). Ratio of slopes for $a_{\mathrm {f}}/a_{\mathrm {raw}}=1.502$, with corresponding half-lives’ ratio: 0.666. The slope of the regression $\log (\mathrm {cumulative~confirmed})-\log (\mathrm {cumulative~tested})\sim time$ is $-0.017 (-0.018,-0.016)$ corresponding to a half-life (in days) of 40.674(38.280, 43.387). The data for the dates 28th–30th March are removed for the regression analysis

**Fig. 12:**
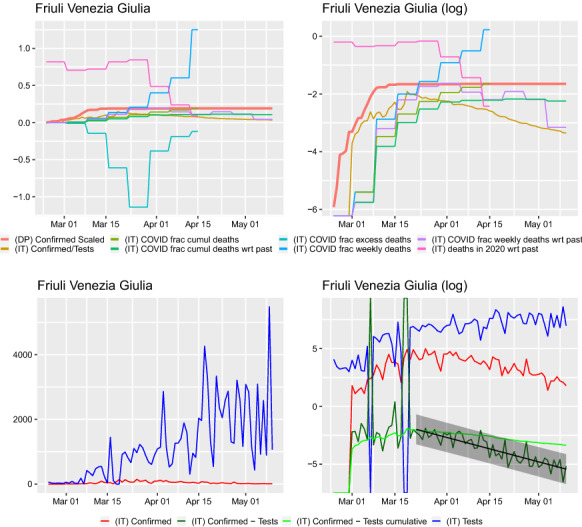
Comparison of curves for Friuli Venezia Giulia region. Left: *y*–axis on normal scale, right: on logarithmic scale. Regression line shown for $\log (\mathrm {daily~confirmed})-\log (\mathrm {daily~tested})\sim time$ with $95\%$ prediction band. Slope of regression with $95\%$ confidence interval: $a_{\mathrm {f}}=-0.070 (-0.082,-0.058)$, this corresponds to a half-life (in days) of 9.885(8.456, 11.894). The slope of the regression $\log (\mathrm {daily~confirmed})\sim time$ is $a_{\mathrm {raw}}=-0.052 (-0.061,-0.043)$ corresponding to a half-life (in days) of 13.315(11.352, 16.098). The slope of the regression $\log (\mathrm {daily~tests})\sim time$ is 0.018(0.007, 0.025) corresponding to a doubling time (in days) of 38.365(27.562, 103.079). Ratio of slopes for $a_{\mathrm {f}}/a_{\mathrm {raw}}=1.347$, with corresponding half-lives’ ratio: 0.742. The slope of the regression $\log (\mathrm {cumulative~confirmed})-\log (\mathrm {cumulative~tested})\sim time$ is $-0.028 (-0.029,-0.027)$ corresponding to a half-life (in days) of 24.866(24.101, 25.680)

**Fig. 13:**
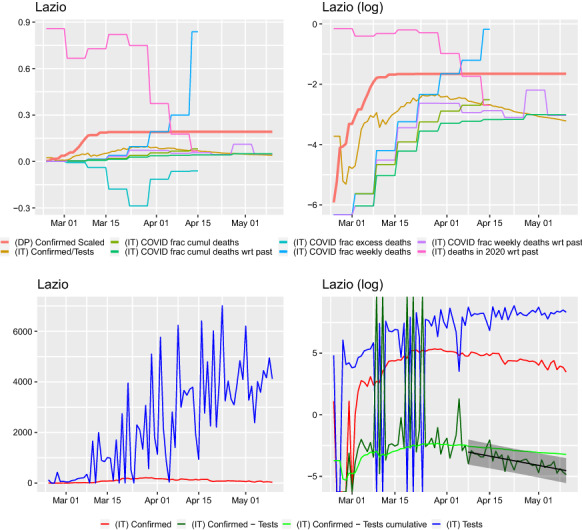
Comparison of curves for Lazio region. Left: *y*–axis on normal scale, right: on logarithmic scale. Regression line shown for $\log (\mathrm {daily~confirmed})-\log (\mathrm {daily~tested})\sim time$ with $95\%$ prediction band. Slope of regression with $95\%$ confidence interval: $a_{\mathrm {f}}= -0.047 (-0.064,-0.030)$, this corresponds to a half-life (in days) of 14.740(10.854, 22.960). The slope of the regression $\log (\mathrm {daily~confirmed})\sim time$ is $a_{\mathrm {raw}}=-0.039 (-0.047,-0.032)$ corresponding to a half-life (in days) of 17.554(14.679, 21.828). The slope of the regression $\log (\mathrm {daily~tests})\sim time$ is $0.008 (-0.007,0.017)$. Ratio of slopes for $a_{\mathrm {f}}/a_{\mathrm {raw}}=1.191$, with corresponding half-lives’ ratio: 0.840. The slope of the regression $\log (\mathrm {cumulative~confirmed})-\log (\mathrm {cumulative~tested})\sim time$ is $-0.021 (-0.022,-0.020)$ corresponding to a half-life (in days) of 32.935(32.015, 33.910)

**Fig. 14:**
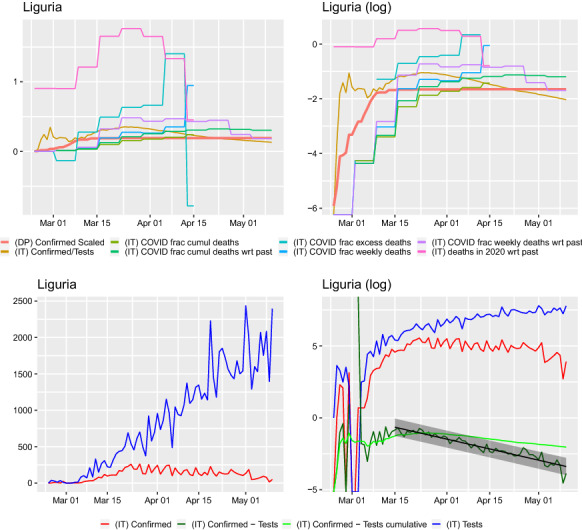
Comparison of curves for Liguria region. Left: *y*–axis on normal scale, right: on logarithmic scale. Regression line shown for $$\log (\mathrm {daily~confirmed})-\log (\mathrm {daily~tested})\sim time$$ with $$95\%$$ prediction band. Slope of regression with $$95\%$$ confidence interval: $$a_{\mathrm {f}}= -0.049 (-0.053,-0.044)$$, this corresponds to a half-life (in days) of 14.217(13.002, 15.682). The slope of the regression $$\log (\mathrm {daily~confirmed})\sim time$$ is $$a_{\mathrm {raw}}=-0.016 (-0.023,-0.010)$$ corresponding to a half-life (in days) of 42.208(29.848, 72.036). The slope of the regression $$\log (\mathrm {daily~tests})\sim time$$ is 0.032(0.028, 0.045) corresponding to a doubling time (in days) of 21.437(15.371, 24.575). Ratio of slopes for $$a_{\mathrm {f}}/a_{\mathrm {raw}}=2.969$$, with corresponding half-lives’ ratio: 0.337. The slope of the regression $$\log (\mathrm {cumulative~confirmed})-\log (\mathrm {cumulative~tested})\sim time$$ is $$ -0.018 (-0.020,-0.017)$$ corresponding to a half-life (in days) of 37.641(34.775, 41.024)

**Fig. 15:**
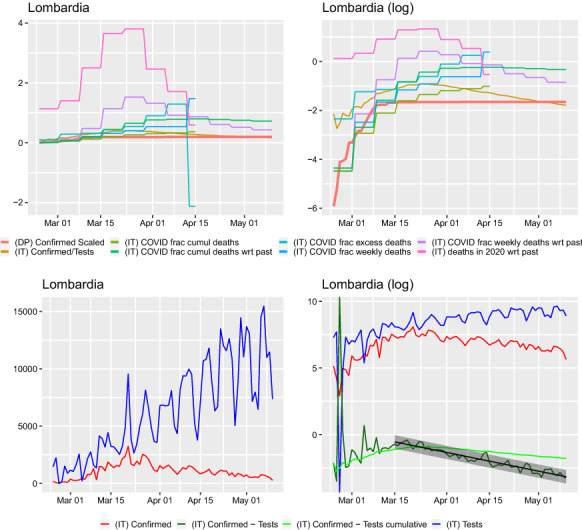
Comparison of curves for Lombardia region. Left: *y*–axis on normal scale, right: on logarithmic scale. Regression line shown for $\log (\mathrm {daily~confirmed})-\log (\mathrm {daily~tested})\sim time$ with $95\%$ prediction band. Slope of regression with $95\%$ confidence interval: $a_{\mathrm {f}}=-0.047 (-0.051,-0.043)$, this corresponds to a half-life (in days) of 14.829(13.712, 16.144). The slope of the regression $\log (\mathrm {daily~confirmed})\sim time$ is $a_{\mathrm {raw}}=-0.025 (-0.029,-0.021)$ corresponding to a half-life (in days) of 27.656(23.961, 32.698). The slope of the regression $\log (\mathrm {daily~tests})\sim time$ is 0.022(0.016, 0.030) corresponding to a doubling time (in days) of 31.970(23.280, 42.506). Ratio of slopes for $a_{\mathrm {f}}/a_{\mathrm {raw}}=1.865$, with corresponding half-lives’ ratio: 0.536. The slope of the regression $\log (\mathrm {cumulative~confirmed})-\log (\mathrm {cumulative~tested})\sim time$ is $-0.016 (-0.018,-0.015)$ corresponding to a half-life (in days) of 42.525(39.386, 46.208)

**Fig. 16:**
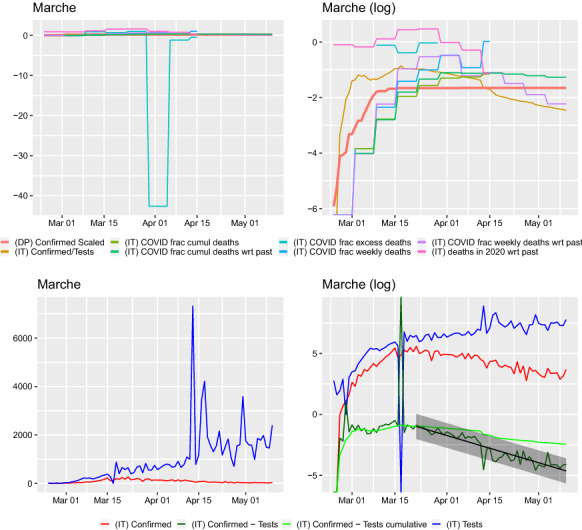
Comparison of curves for Marche region. Left: *y*–axis on normal scale, right: on logarithmic scale. Regression line shown for $\log (\mathrm {daily~confirmed})-\log (\mathrm {daily~tested})\sim time$ with $95\%$ prediction band. Slope of regression with $95\%$ confidence interval: $a_{\mathrm {f}}=-0.074 (-0.084,-0.065)$, this corresponds to a half-life (in days) of 9.304(8.256, 10.657). The slope of the regression $\log (\mathrm {daily~confirmed})\sim time$ is $a_{\mathrm {raw}}=-0.046 (-0.052,-0.040)$ corresponding to a half-life (in days) of 14.990(13.307, 17.161). The slope of the regression $\log (\mathrm {daily~tests})\sim time$ is 0.028(0.019, 0.020) corresponding to a doubling time (in days) of 24.531(35.397, 35.914). Ratio of slopes for $a_{\mathrm {f}}/a_{\mathrm {raw}}=1.611$, with corresponding half-lives’ ratio: 0.621. The slope of the regression $\log (\mathrm {cumulative~confirmed})-\log (\mathrm {cumulative~tested})\sim time$ is $-0.036 (-0.037,-0.034)$ corresponding to a half-life (in days) of 19.519(18.515, 20.639)

**Fig. 17:**
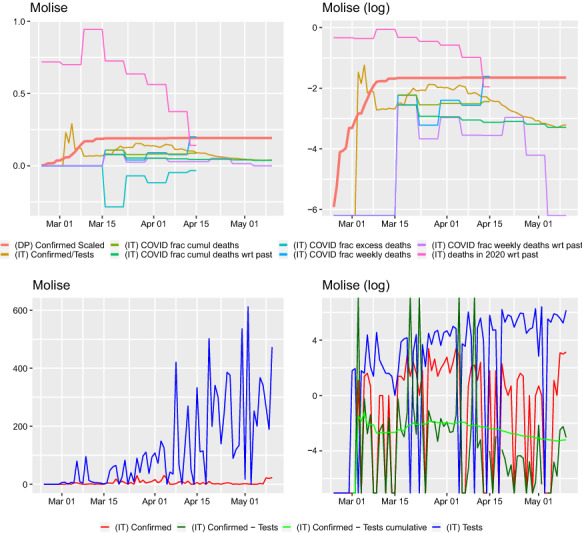
Comparison of curves for Molise region. Left: *y*–axis on normal scale, right: on logarithmic scale

**Fig. 18:**
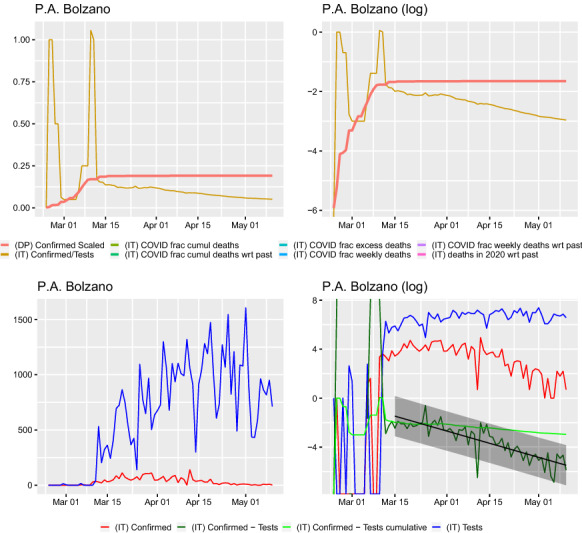
Comparison of curves for P.A. Bolzano region. Left: *y*–axis on normal scale, right: on logarithmic scale. Top row: scaling with respect to population death tolls not presented as P. A. Bolzano is merged with P. A. Trento in deaths date provided by ISTAT. Regression line shown for $$\log (\mathrm {daily~confirmed})-\log (\mathrm {daily~tested})\sim time$$ with $$95\%$$ prediction band. Slope of regression with $$95\%$$ confidence interval: $$a_{\mathrm {f}}=-0.072 (-0.084,-0.059)$$, this corresponds to a half-life (in days) of 9.681(8.246, 11.720). The slope of the regression $$\log (\mathrm {daily~confirmed})\sim time$$ is $$a_{\mathrm {raw}}= -0.061 (-0.074,-0.047)$$ corresponding to a half-life (in days) of 11.385(9.321, 14.623). The slope of the regression $$\log (\mathrm {daily~tests})\sim time$$ is 0.011(0.004, 0.040) corresponding to a doubling time (in days) of 64.662(17.302, 191.080). Ratio of slopes for $$a_{\mathrm {f}}/a_{\mathrm {raw}}=1.176$$, with corresponding half-lives’ ratio: 0.850. The slope of the regression $$\log (\mathrm {cumulative~confirmed})-\log (\mathrm {cumulative~tested})\sim time$$ is $$ -0.018 (-0.019,-0.018)$$ corresponding to a half-life (in days) of 37.589(35.994, 39.332)

**Fig. 19:**
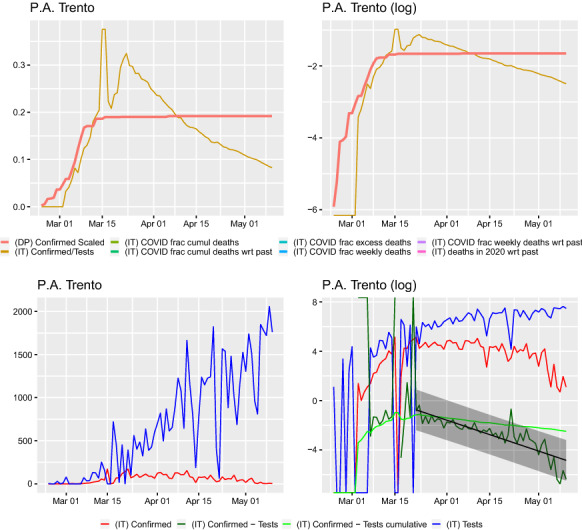
Comparison of curves for P.A. Trento region. Left: *y*–axis on normal scale, right: on logarithmic scale. Top row: scaling with respect to population death tolls not presented as P. A. Bolzano is merged with P. A. Trento in deaths date provided by ISTAT. Regression line shown for $\log (\mathrm {daily~confirmed})-\log (\mathrm {daily~tested})\sim time$ with $95\%$ prediction band. Slope of regression with $95\%$ confidence interval: $a_{\mathrm {f}}= -0.083 (-0.099,-0.068)$, this corresponds to a doubling time (in days) of 8.308(7.033, 10.148). The slope of the regression $\log (\mathrm {daily~confirmed})\sim time$ is $a_{\mathrm {raw}}=-0.055 (-0.069,-0.040)$ corresponding to a half-life (in days) of 12.714(10.010, 17.420). The slope of the regression $\log (\mathrm {daily~tests})\sim time$ is 0.029(0.017, 0.031) corresponding to a doubling time (in days) of 23.975(22.069, 39.784). Ratio of slopes for $a_{\mathrm {f}}/a_{\mathrm {raw}}=1.530$, with corresponding half-lives’ ratio: 0.653. The slope of the regression $\log (\mathrm {cumulative~confirmed})-\log (\mathrm {cumulative~tested})\sim time$ is $-0.027 (-0.028,-0.027)$ corresponding to a half-life (in days) of 25.537(25.139, 25.948)

**Fig. 20:**
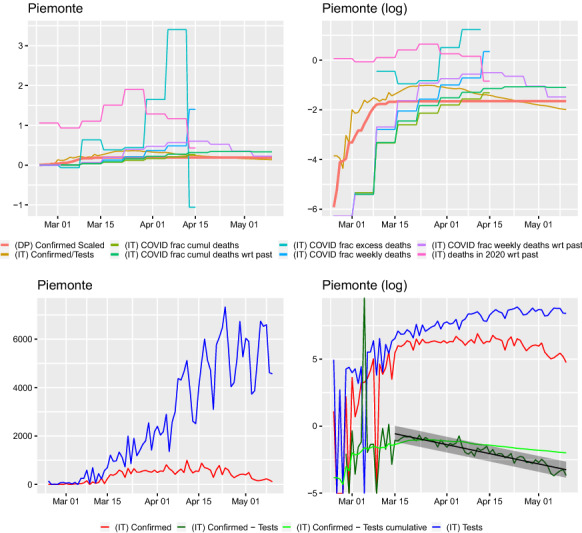
Comparison of curves for Piemonte region. Left: *y*–axis on normal scale, right: on logarithmic scale. Regression line shown for $\log (\mathrm {daily~confirmed})-\log (\mathrm {daily~tested})\sim time$ with $95\%$ prediction band. Slope of regression with $95\%$ confidence interval: $a_{\mathrm {f}}=-0.048 (-0.052,-0.043)$, this corresponds to a half-life (in days) of 14.451(13.207, 15.954). The slope of the regression $\log (\mathrm {daily~confirmed})\sim time$ is $a_{\mathrm {raw}}=-0.013 (-0.019,-0.006)$ corresponding to a half-life (in days) of 53.716(35.637, 109.020). The slope of the regression $\log (\mathrm {daily~tests})\sim time$ is 0.035(0.030, 0.052) corresponding to a doubling time (in days) of 19.769(13.415, 23.179). Ratio of slopes for $a_{\mathrm {f}}/a_{\mathrm {raw}}=3.717$, with corresponding half-lives’ ratio: 0.269. The slope of the regression $\log (\mathrm {cumulative~confirmed})-\log (\mathrm {cumulative~tested})\sim time$ is $-0.018 (-0.019,-0.016)$ corresponding to a half-life (in days) of 39.243(35.548, 43.796)

**Fig. 21:**
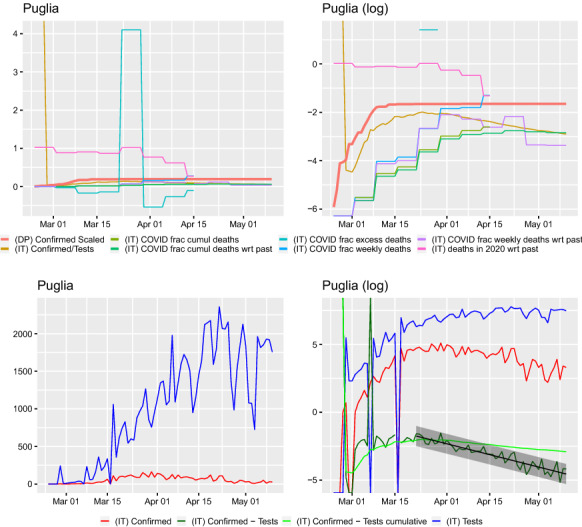
Comparison of curves for Puglia region. Left: *y*–axis on normal scale, right: on logarithmic scale. Regression line shown for $\log (\mathrm {daily~confirmed})-\log (\mathrm {daily~tested})\sim time$ with $95\%$ prediction band. Slope of regression with $95\%$ confidence interval: $a_{\mathrm {f}}=-0.057 (-0.064,-0.050)$, this corresponds to a half-life (in days) of 12.157(10.864, 13.800). The slope of the regression $\log (\mathrm {daily~confirmed})\sim time$ is $a_{\mathrm {raw}}=-0.041 (-0.050,-0.032)$ corresponding to a half-life (in days) of 16.877(13.840, 21.622). The slope of the regression $\log (\mathrm {daily~tests})\sim time$ is 0.016(0.010, 0.033) corresponding to a doubling time (in days) of 43.470(20.965, 68.915). Ratio of slopes for $a_{\mathrm {f}}/a_{\mathrm {raw}}=1.388$, with corresponding half-lives’ ratio: 0.720. The slope of the regression $\log (\mathrm {cumulative~confirmed})-\log (\mathrm {cumulative~tested})\sim time$ is $-0.020 (-0.021,-0.019)$ corresponding to a half-life (in days) of 34.395(33.273, 35.594)

**Fig. 22:**
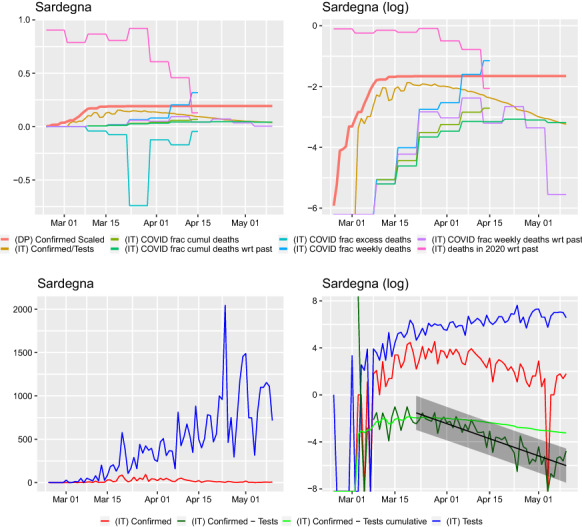
Comparison of curves for Sardegna region. Left: *y*–axis on normal scale, right: on logarithmic scale. Regression line shown for $\log (\mathrm {daily~confirmed})-\log (\mathrm {daily~tested})\sim time$ with $95\%$ prediction band. Slope of regression with $95\%$ confidence interval: $a_{\mathrm {f}}=-0.092 (-0.105,-0.078)$, this corresponds to a half-life (in days) of 7.573(6.599, 8.884). The slope of the regression $\log (\mathrm {daily~confirmed})\sim time$ is $a_{\mathrm {raw}}=-0.058 (-0.072,-0.044)$ corresponding to a half-life (in days) of 11.902(9.603, 15.649). The slope of the regression $\log (\mathrm {daily~tests})\sim time$ is 0.031(0.023, 0.026) corresponding to a doubling time (in days) of 22.193(26.372, 30.382). Ratio of slopes for $a_{\mathrm {f}}/a_{\mathrm {raw}}=1.572$, with corresponding half-lives’ ratio: 0.636. The slope of the regression $\log (\mathrm {cumulative~confirmed})-\log (\mathrm {cumulative~tested})\sim time$ is $-0.030 (-0.031,-0.028)$ corresponding to a half-life (in days) of 23.310(22.147, 24.602)

**Fig. 23:**
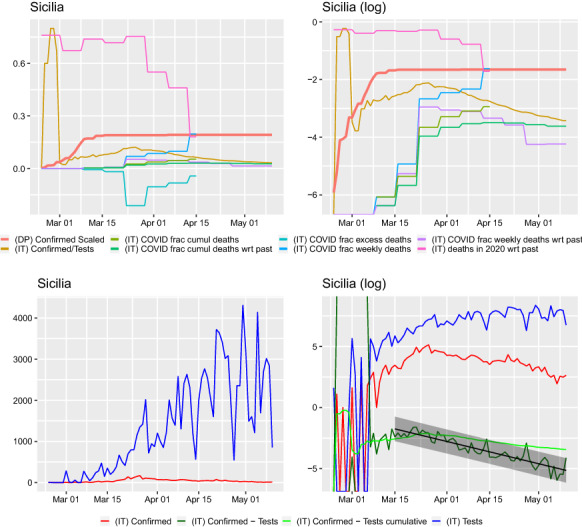
Comparison of curves for Sicilia region. Left: *y*–axis on normal scale, right: on logarithmic scale. Regression line shown for $\log (\mathrm {daily~confirmed})-\log (\mathrm {daily~tested})\sim time$ with $95\%$ prediction band. Slope of regression with $95\%$ confidence interval: $a_{\mathrm {f}}=-0.061 (-0.068,-0.053)$, this corresponds to a half-life (in days) of 11.430(10.156, 13.069). The slope of the regression $\log (\mathrm {daily~confirmed})\sim time$ is $a_{\mathrm {raw}}=-0.030 (-0.038,-0.022)$ corresponding to a half-life (in days) of 22.916(18.243, 30.808). The slope of the regression $\log (\mathrm {daily~tests})\sim time$ is 0.030(0.022, 0.047) corresponding to a doubling time (in days) of 22.803(14.642, 31.528). Ratio of slopes for $a_{\mathrm {f}}/a_{\mathrm {raw}}=2.005$, with corresponding half-lives’ ratio: 0.499. The slope of the regression $\log (\mathrm {cumulative~confirmed})-\log (\mathrm {cumulative~tested})\sim time$ is $-0.023 (-0.026,-0.021)$ corresponding to a half-life (in days) of 29.589(26.719, 33.149)

**Fig. 24:**
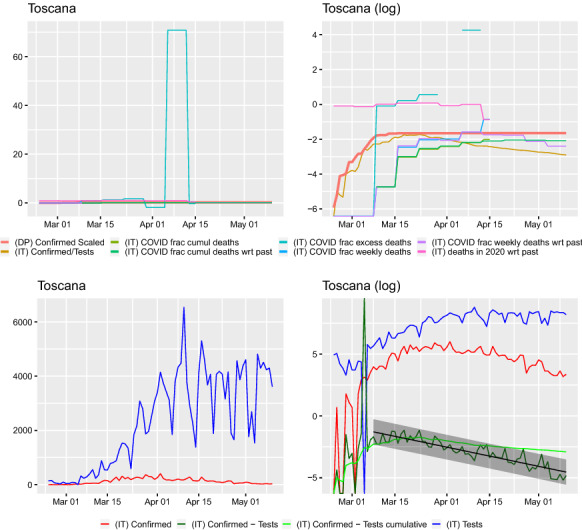
Comparison of curves for Toscana region. Left: *y*–axis on normal scale, right: on logarithmic scale. Regression line shown for $\log (\mathrm {daily~confirmed})-\log (\mathrm {daily~tested})\sim time$ with $95\%$ prediction band. Slope of regression with $95\%$ confidence interval: $a_{\mathrm {f}}=-0.052 (-0.058,-0.045)$, this corresponds to a half-life (in days) of 13.430(11.920, 15.379). The slope of the regression $\log (\mathrm {daily~confirmed})\sim time$ is $a_{\mathrm {raw}}=-0.020 (-0.029,-0.011)$ corresponding to a half-life (in days) of 34.753(23.956, 63.267). The slope of the regression $\log (\mathrm {daily~tests})\sim time$ is 0.032(0.024, 0.059) corresponding to a doubling time (in days) of 21.890(11.681, 28.539). Ratio of slopes for $a_{\mathrm {f}}/a_{\mathrm {raw}}=2.588$, with corresponding half-lives’ ratio: 0.386. The slope of the regression $\log (\mathrm {cumulative~confirmed})-\log (\mathrm {cumulative~tested})\sim time$ is $-0.017 (-0.019,-0.015)$ corresponding to a half-life (in days) of 40.971(36.036, 47.472)

**Fig. 25:**
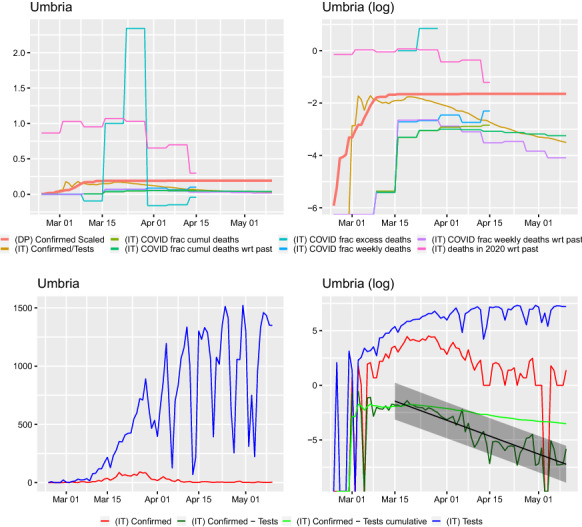
Comparison of curves for Umbria region. Left: *y*–axis on normal scale, right: on logarithmic scale. Regression line shown for $$\log (\mathrm {daily~confirmed})-\log (\mathrm {daily~tested})\sim time$$ with $$95\%$$ prediction band. Slope of regression with $$95\%$$ confidence interval: $$a_{\mathrm {f}}=-0.103 (-0.116,-0.090)$$, this corresponds to a half-life (in days) of 6.720(5.959, 7.704). The slope of the regression $$\log (\mathrm {daily~confirmed})\sim time$$ is $$a_{\rm raw} = -0.079 (-0.093,-0.065)$$ corresponding to a half-life (in days) of 8.810(7.478, 10.720). The slope of the regression $$\log (\mathrm {daily~tests})\sim time$$ is 0.022(0.011, 0.033) corresponding to a doubling time (in days) of 32.197(21.311, 61.463). Ratio of slopes for $$a_{\mathrm {f}}/a_{\mathrm {raw}}=1.311$$, with corresponding half-lives’ ratio: 0.763. The slope of the regression $$\log (\mathrm {cumulative~confirmed})-\log (\mathrm {cumulative~tested})\sim time$$ is $$ -0.035 (-0.036,-0.033)$$ corresponding to a half-life (in days) of 19.892(19.118, 20.731)

**Fig. 26:**
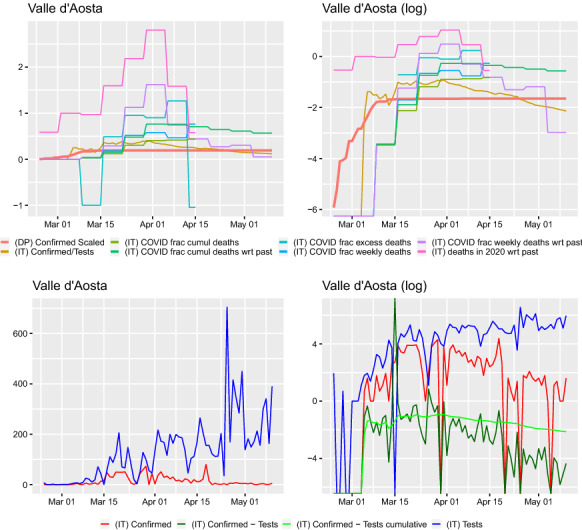
Comparison of curves for Valle d’Aosta region. Left: *y*–axis on normal scale, right: on logarithmic scale

**Fig. 27:**
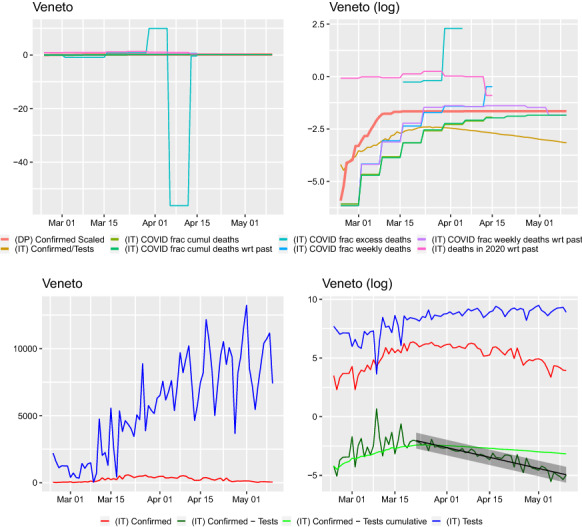
Comparison of curves for Veneto region. Left: *y*–axis on normal scale, right: on logarithmic scale. Regression line shown for $\log (\mathrm {daily~confirmed})-\log (\mathrm {daily~tested})\sim time$ with $95\%$ prediction band. Slope of regression with $95\%$ confidence interval: $a_{\mathrm {f}}=-0.059 (-0.065,-0.053)$, this corresponds to a half-life (in days) of 11.693(10.592, 13.049). The slope of the regression $\log (\mathrm {daily~confirmed})\sim time$ is $a_{\mathrm {raw}}=-0.047 (-0.054,-0.040)$ corresponding to a half-life (in days) of 14.843(12.879, 17.513). The slope of the regression $\log (\mathrm {daily~tests})\sim time$ is 0.013(0.007, 0.026) corresponding to a doubling time (in days) of 55.100(26.467, 95.637). Ratio of slopes for $a_{\mathrm {f}}/a_{\mathrm {raw}}=1.269$, with corresponding half-lives’ ratio: 0.788. The slope of the regression $\log (\mathrm {cumulative~confirmed})-\log (\mathrm {cumulative~tested})\sim time$ is $-0.016 (-0.017,-0.016)$, corresponding to a half-life (in days) of 42.325(40.947, 43.799)

## Appendix A: Curves for regions of Italy

(See Figs. 7, 8, 9, 10, 11, 12, 13, 14, 15, 16, 17, 18, 19, 20, 21, 22, 23, 24, 25, 26 and 27)

## Appendix B: Death tolls for regions of Italy

(See Figs. 28, 29, 30, 31 and 32)

## Footnotes

1 Initially, the Veneto region blanked tested a significant part of the population, while Lombardy did not (private communication with Marco Picariello and Paola Aliani).

2 https://www.istat.it/it/archivio/240401.

3 A similar graphical analysis for appeared in [8, 10, 27].

4 https://www.ons.gov.uk/peoplepopulationandcommunity/birthsdeathsandmarriages/deaths/articles/highestnumberofexcesswinterdeathssince19992000/2015-11-25.

5 Private communication with Marco Picariello and Paola Aliani.

6 https://cran.r-project.org/web/packages/COVID19/.

7 https://github.com/CSSEGISandData/COVID-19.

8 https://data.worldbank.org/.

9 https://www.cia.gov/library/publications/resources/the-world-factbook/fields/343rank.html.

10 https://github.com/pcm-dpc/COVID-19.

11 https://www.istat.it/en/population-and-households?data-and-indicators.

12 https://www.bfs.admin.ch/bfs/en/home/statistics/regional-statistics/regional-portraits-key-figures/cantons/data-explanations.html.

13 https://github.com/openZH/covid_19.

14 https://covid19datahub.io.
